# Portable and wide-range solid-state transmission densitometer for quality control in film radiography

**DOI:** 10.4103/0971-6203.62135

**Published:** 2010

**Authors:** Javier Morales Aramburo, Sigifredo Solano Gonzalez, Jorge Toledo Toledo

**Affiliations:** Physics Department, National University of Colombia, Calle 59A No 63-20 - Núcleo El Volador, Medellín – Antioquia, Colombia, Medellín-Colombia

**Keywords:** Densitometer, junction temperature, light-emitting diode, optical density

## Abstract

In biology, materials science, radiography quality control or film dosimetry in radiotherapy, a transmission densitometer is useful for measurements of optical density. The design proposed here is oriented to quality control in radiographic films. The instrument described here utilizes low-cost solid-state devices and is easy to construct. The use of 1-watt white light-emitting diode in this densitometer enables low power consumption and a cold light source. Moreover, the instrument does not need a reference light, which results in decreasing the number of parts and reducing the overall size of the apparatus.

## Introduction

Organizations like the International Atomic Energy Agency (IAEA) and the International Radiation Protection Association (IRPA) supported by World Health Organization (WHO) establish quality assurance programs in diagnostic radiology and radiotherapy, within which densitometry is an important part of quality control of image-forming systems with X-rays and dosimetry. In densitometry the degree of the film blackening is measured by determining the optical density (OD) by a densitometer. OD is defined as the ratio of light incident on the film to the light transmitted.

(1) $$\mathrm{OD}\;=\;\log_{10}\left(\frac{I_{0}}{I_{i}}\right)$$

**Figure 1:**
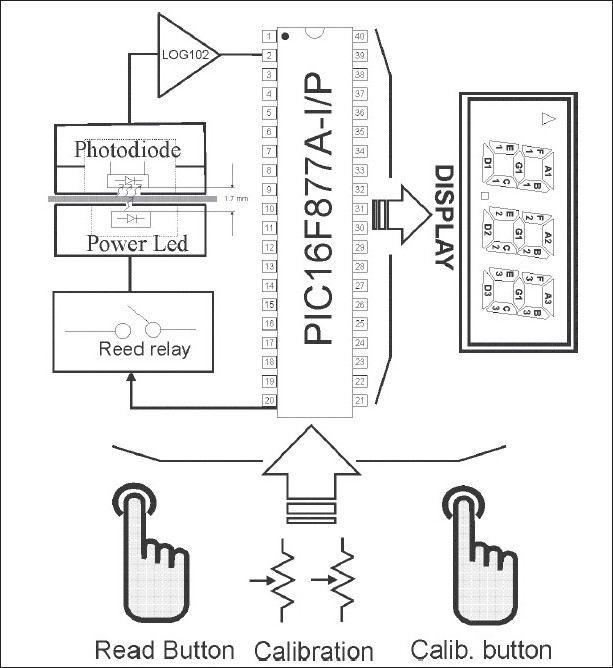
System framework of the transmission densitometer

The range of OD measured by the instrument is between 0.15 and 4.0 with an uncertainty of ±0.02 OD. Even other instruments have been constructed for this purpose, but the technology is old[1] or they do not cover a wide range of optical density.[2] In other fields, a similar technique has been used with good results as those presented here.[3] An overview of the system framework of the transmission densitometer is shown in Figure 1. The instrument consists of a light source, viz., light-emitting diode (LED), a tiny aperture through which the light is directed; and a light detector (photodiode) to measure the light intensity transmitted to the film. The acquisition and signal conditioning are achieved by implementing a circuit with the well-known integrated logarithmic amplifier LOG102. A microcontroller performs the calculation of optical density and its display in a low-power consumption liquid crystal display (LCD). The calibration is achieved from two variable voltage dividers; the values of these voltages are used like an offset to adjust the values of optical density displayed to the values of a calibration density strip tablet with the National Institute of Standards and Technology (NIST) traceability.

## Materials and Methods

### Signal acquisition

**Figure 2:**
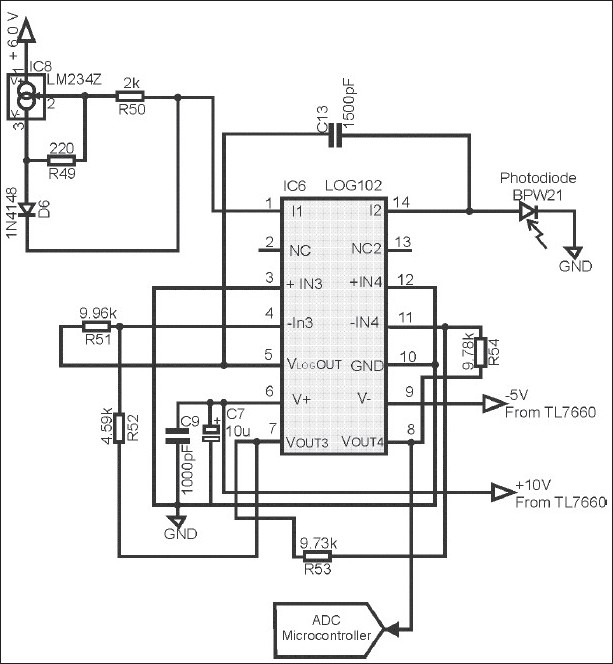
Current source LM234Z and the Log102

The heart of the instrument is the logarithmic amplifier LOG102 Figure 2. This amplifier is able to measure the 5 decades of the current (i_2_) that produces the photodiode with optical densities from 0 to 4. The light detector comprises a photodiode with spectral response in the visible range connected to the logarithmic amplifier. This design takes advantage of the linear (light intensity/diode current) relationship obtainable in the short circuit mode of operation of the photodiode. Moreover, this mode of operation gives the short response time required for fast film scanning.

### Current and voltage sources

The logarithmic amplifier gives a voltage response proportional to the ratio of the current i_1_ to the i_2_, where i_1_ is a current set to 500 *μ*A with a 3-terminal adjustable current source. This current adjusts the voltage range of the logarithmic amplifier to a maximum value in the case of the 4.00 OD suitable for A/D conversion; and in the case of measurement without film, a minimum voltage that will be displayed like a 0.00 OD in the LCD. The instrument takes power directly from four AA batteries, which produce 6 volts, 4.5 volts for the microcontroller; and the other voltages required for the operational amplifiers and a negative voltage supply are obtained from a complementary metal-oxide-semiconductor (CMOS) switched-capacitor voltage converter (TL7660). The current source for the LED was constructed with a linear voltage regulator; it is inexpensive, and due to the feedback and an internal voltage reference of 1.25 V, it keeps a constant current in the LED.

### Calibration and measurements

With all the blocks working together, the next step is the calibration. The reference voltage for the A/D conversion is obtained from a 2.5-volt precision voltage reference integrated circuit. From its output are connected two variable voltage dividers. With high input impedance voltage followers, the two voltages from the two voltage dividers are carried to the analog-to-digital converter of the microcontroller. The logarithmic amplifier gives a curve of response in the range of the OD values measured, which may be adjusted to the calibrated values of the density strip in each step by a linear relation. The constants of this simple linear relation (2) are set by means of variable voltage dividers. In the calibration of the instrument, one of the potentiometers is used to adjust an offset voltage, represented by α, and the other is used to set the constant β. Finally the microcontroller obtains the optical density value from the equation (2), where V_LOG102_ is the voltage from the logarithmic amplifier.

(2) $$\mathrm{OD}=-\alpha +\beta \times V_{\mathrm{LOG102}}$$

### Mechanical and thermal noise

An important source of noise found in the measurement is the mechanical structure. The photodiode and the LED should be aligned, and only up and down movement is allowed. The distance between LED and photodiode should always be 1.7 mm. Light from the LED should pass through a pinhole of radius 1 mm, and the cup of the LED should be removed because the nearest distance between LED and photodiode is required by the use of little forward current. The thermal noise comes from the increment of the junction temperature in the LED diode junction by the forward current, causing relative photometric output changes,[4] as well as loss of calibration. The current used to ensure the accuracy of the measurements is only 25 mA, and the time by which the LED is turned on is about 4 seconds, to avoid problems of accuracy due to the increment in the junction temperature. The white LED used is a neutral white LED.

## Results and Discussions

The calibration standard for optical density values was an AGFA - STRUCTURIX–calibrated step tablet with 14 steps, identification number 6414163, and NIST traceability. The uncertainty of this strip tablet is ±0.006 OD, and the measurements made with this instrument in each step of the tablet give a value with a maximum deviation of ±0.02 OD with respect to the value in the calibration tablet [Table 1]. The repeatability of the measurements was achieved by solving the problems related to the different sources of noise. The instrument provides accuracy in measurements with respect to the values of the calibration standard; and in cases when a deviation was observed, the difference with respect to the calibration standard was not higher than ±0.02 OD.

**Table 1:** Measurements made with the densitometer in each step of the tablet

*OD measured with the instrument*	*OD values in the calibrated step tablet*	*Error = x_measured_ − x_true_*
0.00	0.000	0.000
0.13	0.146	0.016
0.30	0.296	−0.004
0.59	0.588	−0.002
0.91	0.903	−0.007
1.21	1.200	−0.010
1.50	1.500	0.000
1.82	1.810	−0.010
2.10	2.090	−0.010
2.40	2.390	−0.010
2.69	2.680	−0.010
2.98	2.980	0.000
3.31	3.300	−0.010
3.58	3.590	0.010
3.86	3.880	0.020
4.16	4.180	0.020

## Conclusions

A simple method using a white-light LED and photodiode for optical density measurement of radiographic films was developed. The calibration system is straightforward and provides stability in measurements because of the circuit used to maintain the values of the constants of calibration with a precision voltage reference integrated circuit. Excellent repeatability was obtained, and the uncertainty in the measurements was found to be similar to that obtained by using commercial densitometers (±0.02 OD). This type of densitometer can be used easily in many radio-diagnostic and radiotherapy centers.
